# To assess the level of knowledge, attitude, and practice of communication among ICU trainees across India, TALK ICU SURVEY

**DOI:** 10.1186/s12909-025-07977-z

**Published:** 2025-10-17

**Authors:** Amarja Ashok Havaldar, Harshavardhini C P

**Affiliations:** Department of Critical Care Medicine, St. John’s Medical College Hospital, Bangalore, 560034 India

**Keywords:** Doctor-patient relation, Communication, Critical care, Curriculum, Intensive care unit

## Abstract

**Background:**

Effective communication skills are the key component in the doctor-patient relationship. We aimed to assess the level of knowledge of communication among the ICU trainees across India.

**Methods:**

We conducted a nationwide survey. The survey comprised diverse aspects like breaking bad news, taking informed consent, communicating a medical error, reasons for multidisciplinary conflicts, and end-of-life care (EOLC) policies.

**Results:**

A total of 146 responses were analysed. The predominant age group was 31–40 yrs. (72.60%) with female preponderance (63.7%). Most trainees had 1–5 years of work experience, had post-graduation in anesthesiology, and enrolled for the Indian Diploma in Critical Care Medicine. Only around 50% had formal communication training. Barriers to counselling were the education level of the family (78.76%), language (43.15%), burnout of the treating doctor (43.15%), and the severity of illness (41.09%). The majority were aware of the EOLC policy (59.58%) and felt this was the need of the hour (96.57%). The SPIKES (Setting up, Perception, Invitation, Knowledge, Emotions with Empathy, and Strategy or Summary) protocol was mentioned only by 13.98% of trainees as a strategy for communication. Trainees who had received formal training in communication had more experience in communicating a medical error and had less difficulty in breaking bad news as compared to trainees with no formal training in communication (*p* < 0.05). Reasons for interdisciplinary conflicts among doctors included communication and knowledge gaps, ego, and differences of opinion. The trainees suggested strategies for improving communication skills, including formal training, real-time communication experience under supervision, the use of simulation, and adherence to standard operating procedures.

**Conclusion:**

The current survey revealed that trainees lacked adequate knowledge and skills in communication. These findings can help design the training curriculum focused on communication skills.

**Supplementary Information:**

The online version contains supplementary material available at 10.1186/s12909-025-07977-z.

## Introduction

A doctor-patient bond is built on the foundation of effective communication. Communication is based on the source, message, and receiver [1]. The physician is the source, the clinical condition is the message, and the patient or family is the receiver. Effective communication is built when the source and the receiver attribute the same meaning to the given message [2]. Unfortunately, most communication skills are learned non-formally through the process of observing and simulating words and gestures. This creates a huge lacuna in the system.

In current-day critical care practices, there are many stakeholders in patient care. It’s a big team of doctors, nurses, physiotherapists, dieticians, medical social workers, and many more. A survey from India showed family satisfaction was associated with addressing patients’ symptoms, the approach of the ICU staff towards patient relatives, and concern towards patients’ needs and families. An interesting finding was that family satisfaction was not related to good clinical outcomes [3]. Critically ill patients differ from other patients in the hospital because of physical, physiological, and psychological barriers to communication. The next of kin are at an extreme level of stress due to the dynamicity of the course of illness, the logistics burden, and caregiver burnout. A study comparing the American and Indian population showed that relatives of patients in the Indian ICUs had greater anxiety and depression symptoms compared to those in the American cohort. This was associated with the substantial discordance between perceived and objective severity of illness [4]. Death in the ICU is not uncommon. The death can be either respectful with the loved ones around or in a dehumanised atmosphere surrounded by machines [5]. EOLC is a critical decision that is influenced by the ecosystem, culture, law, public policy, institutional policy, physicians attitudes, and social beliefs [6]. Due to moral distress, caregiver burden, economic and logistic hardships, Indian families fail to understand the idea of “good dying” [7]. With the changing landscape of End Of Life Care policies (EOLC), the families can be informed well in advance about the prognosis and overall quality of life of their loved ones [5, 8]. Effective communication can help in reducing anxiety and depression symptoms among family members.

Position statements designed by the Indian Society of Critical Care Medicine (ISCCM) and the Indian Academy of Palliative Care (IAPC) Experts take into account available evidence, ethics, and law. It gives a framework to the treating physician [8].

Violence against medical professionals is as high as 75% according to the Indian Medical Association, and 90% across the globe [9, 10]. The poor communication skills of healthcare workers and staff are one of the potential causes of violence against doctors [9, 10]. A survey conducted among trained intensivists across India showed that they could effectively deliver bad news, but they failed to attend to the emotional needs of the family [11].

When the communication transitions from daily updates to more complex scenarios of EOLC, several barriers to effective communication are encountered. The designed survey focuses on the knowledge, attitudes, and practices of ICU trainees regarding communication skills. This can be the first step towards making conscious efforts to break barriers and build a healthy relationship between doctors, patients, and next of kin.

## Methods

We obtained institutional ethics committee (IEC185/2023) approval. We conducted an online survey between 8 September 2023 and 16 February 2024 across India. All the procedures were followed in accordance with the ethical standards of the responsible committee on human experimentation (institutional or regional) and with the Helsinki Declaration of 1975. The target population was ICU trainees who had finished at least > 3 months of training. Trainees of Doctorate of Medicine, Doctorate of National Board, Indian Diploma in Critical Care Medicine, Indian Fellowship in Critical Care Medicine, and other post-doctoral critical care fellowship programs were included.

### Designing and validation of the questionnaire

We designed a questionnaire focused on various aspects of ICU communication, such as daily updates, barriers to counselling, breaking bad news, informed consent, and EOLC. The survey included an initial section of demographic details followed by understanding communication practices, daily updates, barriers to counselling, breaking bad news, informed consent, medical error, EOLC, and the trainee’s perspective about family satisfaction.

There were binary, multiple-choice, and open-ended questions. The open-ended questions were strategies used for daily communication, difficulties faced during breaking bad news, reasons for interdisciplinary conflicts, changes in doctor-patient relationships post COVID-19, and lastly, suggestions from trainees for improving communication skills (Supplementary material-questionnaire).

The survey was sent to 7 critical care experts. The questionnaire was structured to convey a clear meaning and refined after discussion with critical care experts. Construct and content validity checks were performed. To ensure construct validity, questions on barriers to counselling and family satisfaction were carefully designed with multiple choice options, and these were decided based on the thorough available literature for accurate measurement of these concepts. For content validity, the developed tool was evaluated by subject experts. Using the expert validation and suggestions, relevant domains related to communication in the ICU settings were considered for finalizing the tool. After validation, pilot testing was performed.

### Survey administration

The link to the questionnaire was circulated via social media (WhatsApp groups, Facebook, LinkedIn, and emails) regularly over 6 months. Participation in the study was voluntary, and only after providing consent, the study participants could take up the survey. Confidentiality and anonymity were maintained regarding the participants. Responses received were reviewed. Similar responses to all questions, including demographic details, were considered duplicate entries, and only one entry was included in the analysis.

### Sample size

The survey included trainees enrolled in various training courses. We considered an occupancy of around 80% for various courses in India, and the calculated population size was 1,390. The response rate for the survey was estimated to be 10%, and the calculated sample size was 139.

### Statistical analysis

Among the quantitative data, continuous variables were presented as mean and standard deviation, and categorical data as percentages in descriptive statistics. The analysis, based on the type of hospital and formal training in communication received, was performed using the Chi-square test or Fisher’s exact test as applicable.

### Qualitative analysis

In the qualitative analysis, the responses for open-ended questions were analysed using the implicit technique of content analysis. The strategies followed during counselling were assessed using the Kalamazoo Communication Checklist [12, 13]. Nine phrases were chosen from the checklist: builds relationship, open discussion, gathers information, understands perspectives, shares information, reaches agreement, provides closure, empathy, and accurate information. The responses were translated into these phrases and analyzed. Anything apart from the checklist was classified separately. Each phrase was assigned one point, so the total possible score ranged from a minimum of 0 to a maximum of 9.

Difficulties in breaking bad news were broadly classified under the headings related to the doctor, the patient/family, and the patient’s clinical condition. The reasons for interdisciplinary conflicts were categorised as doctor-doctor, doctor-nurse, and doctor-administration, and were analysed accordingly. The influence of the COVID-19 pandemic on the doctor-patient relationship was classified into three categories: yes, no, and maybe. The words used were categorized into positive and negative effects on the relationship, and reasons were enumerated. The suggestions for improvement in communication skills were classified into different teaching and learning methods.

## Results

### Participants' background

A total of 149 responses were received, and 3 were excluded due to duplicate entries. We analysed 146 responses. The response rate, based on the calculated population, was 10.5% (146/1390). The mean age of participants was 33.4(4.68) years, and the majority were in the 31–40 years age group (Table 1). Female preponderance (63.70%) was observed. Trainees were predominantly from private-corporate hospitals, followed by government medical colleges and private non-profit sectors.

**Table 1:** Demographic characteristics

Variables	All (*N* = 146)
Age ¥ (Years)	33.48 (4.68) mean (SD)
< 30	33 (22.60)
31–40	106 (72.60)
> 40	07 (4.79)
Sex	
Male/Female	53 (36.30)/93 (63.70)
Experience	
< 1 year	36 (24.66)
> 1–5 years	90 (61–64)
> 5–10 years	10 (6.85)
> 10 years	10 (6.85)
Speciality	
Anaesthesiology	103 (71.55)
Emergency medicine	7 (4.79)
General medicine	19 (13.01)
Pulmonary medicine	17 (11.64)
Course enrolled	
DM	29 (19.86)
DrNB	49 (33.56)
IDCCM	51 (34.93)
IFCCM	2(1.37)
Fellowship in critical care	15 (10.27)
Duration of training	
> 3 months – 6 months	13 (8.97)
6 months − 1 year	28 (19.31)
> 1–2 years	41 (28.28)
> 2–3 years	55 (37.93)
Others	8 (5.52)
Type of hospital	
Government/medical college	30 (20.55)
Private/corporate hospital	96 (65.75)
Private hospital non-profit	20 (13.70)
No of patients/day	
< 10	17 (11.64)
> 10–20	84(57.53)
> 20–30	25 (17.12)
> 30	20 (13.17)
Working hours/week	
< 48 h	11 (7.53)
> 48 h	135 (92.47)

*n* (% percentages), ¥ mean (Standard deviation) IDCCM Indian diploma in critical care medicine, IFCCM Indian fellowship in critical care medicine, DM Doctorate of medicine, DrNB Doctorate of national board

### Hospital policies and counselling practices

Formal training was received by 72(49.32%) of trainees. Family meetings were routinely attended by 62.33% and 96.58% had the opportunity for independent counselling. (Supplementary Table S1). The audio-visual counselling facilities were available for 70.55% of the participants, and 56.44% were recording counselling as a routine practice (Supplementary Table S1). Bereavement support was available in 46.57%. EOLC policy existed in 59.58% and 60.27% had code white (for violence against medical care providers) policy (Supplementary Table 2).

The analysis, based on the type of hospitals, revealed similar results across the three groups. However, the availability of audio-visual counselling varied across institutions, with less availability in government institutions. Even when available, its usage was lower compared to private-corporate and private-nonprofit institutions. EOLC and code white policy availability also deferred across the institutions (Supplementary table S3).

Trainees who had received formal training in communication had more experience in communicating a medical error and had less difficulty in breaking bad news as compared to the group with no formal training (Supplementary Table S4).

### Barriers in counselling

Major barriers in counselling were the family’s education level (78.76%), burnout of the treating doctor (43.15%), language (43.15%), severity of illness (41.09%), and length of ICU stay (37.67%). Also 11.64% mentioned gender of the communicating doctor as a barrier to counselling (Table 2). Doctor’s perception of the family’s satisfaction was influenced by various factors as mentioned in Table 2.

**Table 2:** Barriers in counselling and factors influencing family satisfaction

Barriers in counselling	Education level of family	115 (78.76)
	Language	63 (43.15)
	Burn out	63 (43.15)
	Severity of illness	60 (41.09)
	Length of ICU stay	55 (37.67)
	Socioeconomic status	50 (34.24)
	Experience of the treating doctor	38 (26.02)
	Gender of the communicating doctor	17 (11.64)
	Other (workload of the doctor)	1 (0.68)
Factors influencing patient family satisfaction	Counselling by senior faculty	116 (79.45)
	Visiting hours	109 (74.65)
	Outcome of patient	109 (74.65)
	Lack of empathy among caregivers	66 (45.20)
	Paramedical staff interaction	64 (43.83)
	Lack of administrative support	56 (38.35)

*n* (% percentage)

### Strategies used during daily counselling

We used the Kalamazoo checklist to evaluate the strategies used for counselling. SPIKES-(Setting up, Perception, Invitation, Knowledge, Emotions with Empathy, and Strategy or Summary) as the keyword was mentioned only by 13.98%. Commonly used strategies were classified into phrases used in the Kalamazoo checklist (Table 3). We did not observe all 9 phrases of the Kalamazoo checklist being mentioned by the trainees.

**Table 3:** Strategies used for counselling

Available responses *n* = 143	Percentage (%)
SPIKES	13.98
Builds relationship	39.1
Opens discussion	16.7
Gathers information	16.08
Understands patient/family perspective	14.6
Shares information	68.5
Reaches agreement	8.39
Provides closure	30.06
Demonstrates empathy	12.58
Communicates accurate information	32.86
**Strategies other than components of SPIKES**	
Simple language	4.19
As per patient Condition	9.79
Affordability of the family	4.19
Documentation	1.39
Multidisciplinary	0.69
Not counselling	1.39
**Counselling in different situations**	
Informed consent: Detailed explanation of all the procedure related complications	Yes 77(52.73) No 4(2.73) Sometimes 22(15.06) Depends upon type of procedure 43(29.45)
Informed consent from patient	Yes 121(82.87) No 25(17.12)
Medical error counselling	Yes 60(41.09) No 86(58.90)

Values are numbers (% percentage)

### Informed consent and medical error

The question related to informed consent for the procedure, 77(52.73%) responded by communicating all the complications associated with the procedure, and 43(29.45%) mentioned that, depending on the type of procedure, complications will be informed (Table 3). Among 146 trainees, 60 (41.09%) had counselled relatives about the medical error (Table 3).

### Difficulties in breaking bad news

Among our participants 39% reported difficulties in breaking bad news. Doctors had difficulties due to aggressive family, moral distress, death declaration, lack of effective communication, shift duties, and limited experience of the doctors. Difficulties due to patient/family factors and disease-related factors are mentioned in Table 4.

**Table 4:** Difficulties encountered in breaking bad news

Doctor	Patient/Family	Disease related
Aggressive Family	Emotional Outburst	Type/severity/prognosis
Moral distress	Lack of understanding	Unanticipated clinical situation
Death Declaration	Unacceptable	Younger age group
Lack Of Effective Communication	Socio-Economic Status	
Shift Duties		
Experience		

### Reasons for interdisciplinary conflicts

The reasons for conflicts between doctors, nurses, and administration are mentioned in Table 5. The reasons for interdisciplinary conflicts between doctors mentioned were communication gap (36.84%), knowledge gap (31.57%), ego (30.70%), difference of opinion (19.29%), burnout (8.77%), lack of team dynamics (7.89%) between doctors, and compartmentalization of care (4.38%) (Table 5).

**Table 5:** Reasons for interdisciplinary conflicts

DOCTOR-DOCTOR (*N* = 114)	
Communication gap	36.84
Knowledge gap	31.57
Ego	30.70
Difference of opinion	19.29
Burn out	8.77
Lack of Team dynamics	7.89
Compartmentalisation of care	4.38
DOCTOR-NURSE (*N* = 20)	
Lack of understanding of critical situation	72.72
Lack of team dynamics	36.36
Burn out	9.09
DOCTOR-ADMINISTRATION (*N* = 31)	
Lack of skilled manpower	48.38
Work environment	32.25
Lack of resources (Equipments)	25.8
Finances	19.35

*n*(% percentage)

The COVID-19 pandemic has influenced doctor-patient interaction. Total responses were 144 for this question. The single word responses or short answers were classified into yes 53(36.80%), no 31(21.52%), and may be 19 (13.19%). The detailed responses [39(27.08%)], we further categorized into different themes as follows. The major changes were in technology 9(23.07%) that influenced the clinical practice. Telecommunication or video counselling were well accepted. However it also led to rise in “Google doctors”. People became aware 7 (17.95%) and recognized 5(12.82%) critical care services. However, there was also rise in mistrust 7(17.95%) towards healthcare. Relatives had mixed experience 7(17.95%) about healthcare workers based on the severity of illness, clinical outcome and finances. There was also effect on mental health like post-traumatic stress disorder 1/39(2.56%) in doctors and patient relatives. Other less common themes were 3(7.69%) improved satisfaction, increased trust, and strengthened doctor–patient relation.

The suggestions for improving communication from the trainees were formal training in communication 44.52%, real-time training methods 12.32%, simulation-based teaching 9.58%, and establishing Standard Operating Procedures (SOP, 9.58%).

## Discussion

The current survey helped to understand various aspects of communication. We had 146 responses from a diverse group across the country. This included participants from government, private non-profit, and corporate sectors. The availability of dedicated counselling rooms, audiovisual facilities, and designated time for patients and family discussions provided insight into the institutional support systems in place to facilitate effective communication. Our results showed that although facilities like audiovisual counselling were available, utilisation differed between different sectors of hospitals (Supplementary Table S3). Use of audiovisual facilities can help in recording the communication and can act as a rich educational resource for teaching communication skills to the trainees [14]. Also, awareness about different policies like EOLC and code white across institutions is necessary (Supplementary Table S3).

We observed that trainees with formal communication training were more comfortable communicating medical errors and breaking bad news, compared to those without formal training (Supplementary Table S4). The study conducted in general surgery residents showed that 37.8% residents had received training in breaking bad news. It also stated that lack of infrastructure and lack of re-enforcement from the seniors about adherence to SPIKES protocol [15]. It was an interview-based observation, as compared to the survey design in our study.

In the current study, SPIKES was mentioned by very few trainees (13.98%) as a strategy for counselling. In a photovoice study, learning communication with families was considered one of the core clinical skills among intensive care residents. The skills associated with effective communication were establishing daily goals, knowing how to socialize, and sharing the goals with family [16]. The SPIKES protocol is widely used in the family meetings [17]. The new protocol, Setting, Perception, Warning call and pause, Information, Clarifying and dealing with Emotions, Strategy and Summary, S-P-w-ICE-S, is introduced to overcome the challenges in using the SPIKES protocol [18]. The additional components included in S-P-w-ICE-S give a framework that helps in dealing with different scenarios in real life.

Among the barriers to counselling, the family’s education level was the most significant barrier. One of the barriers was gender. When we formulated the question, our intent was to determine whether the gender of the treating doctor acts as a barrier. However we did not know how this response is interpreted by the trainees. Detailed interview can help us in understanding this perspective better. A total of 17(11.64%) of trainees mentioned gender as a barrier to counselling. We believe learning effective communication can help in overcoming several barriers.

Total of 39% of our participants had difficulty in breaking bad news. In a study of self-assessment of medical, surgical and emergency residents in breaking the bad news, 46.5% rated themselves to be competent to break the bad news. Challenging aspect of communication was to balance being honest at the same time not to upset the family members. The barriers they faced were aggressive family members, lack of training and experience [19].

To determine the effectiveness of active learning method in communication, an interventional study done by Polivka, et al. comprised of two groups showed residents who were in the interventional group had improvement in the summative assessment score. The evaluation method was Objective Structured Clinical Examination (OSCE) [20]. This again emphasized that formal communication training can be helpful.

The majority of ICUs in India are open ICUs [21]. With the advent of multiple specialties and subspecialties, patient care has moved from the concept of “Family doctor” to the compartmentalization of care. The major reasons for interdisciplinary conflicts in the ICU were ego, difference of opinion, communication, and knowledge gap (Table 5). This shows there is a lack of team dynamics. Deficiencies in administrative support, like a lack of resources (equipment) and skilled manpower, aggravate this issue. We suggest multidisciplinary meeting involving all the stakeholders is necessary as each stakeholder has unique roles and responsibilities. This will ensure holistic patient care. “The Conflicus study”, evaluating prevalence and factors associated with ICU conflicts categorized conflicts as related to behavior and end of life care. Reasons for behavior related conflicts were personal animosity, mistrust, communication gaps, no regular staff meetings and misunderstandings among staffs [22]. Developing a culture of multidisciplinary meetings could ensure a more patient-centered approach [23, 24].

Ninety six percent of our participants felt EOLC policy as the need of the hour. EOLC communication is often perceived as an advanced communication skill leading to hesitation and fear among treating doctors. The article by Ganz, et al. mentions barriers and facilitators for EOLC [25]. The decision making in EOLC includes determining futility, incorporating ethical principles, understanding values and preferences of the patients and also requires knowledge of the law. It requires establishing rapport with the family and understanding their perspectives. Very often several meetings with the legally acceptable representatives may be necessary to arrive at the decision. Using simple checklist or Validate, Assess, Listen, Understand, and Express, “VALUE” mnemonic can help in guiding trainees in introducing EOLC communications early in the illness. One of the article by Randal Curtis, et al. emphasized on importance of teaching palliative care skills and language of the palliative care to the trainees [26]. Hence, strengthening communication skills can ensure a smooth transition from cure to care [27]. The Indian guidelines by ISCCM and IAPC aim to support a humanized, evidence-based, and legally compliant system and also empower trainees as well as faculty in decision making [6].

The COVID-19 pandemic was the eye-opener, which unmasked the deficiencies in health care, including communication. Advancement in technology resulted in the surge of “Google doctors”. This has given rise to new challenges in communication like demystifying myths, rebuilding the trust and treating cyberchondria (anxiety due to alarming or exaggerated online content). Although information is easily available, source and reliability of information is often not verified. This led to mismatch between expectation and outcome, as well as lack of trust in the system. Ultimately manifesting as rising violence against healthcare professionals. This raises fear and concern in aspiring doctors to choose career in medicine. Strengthening communication skills could be one of the key solutions [2].

Communication and interpersonal skills have been imperative since 1997 in medical education. The welcome move in 2015 by the medical council of India led to designing a competency-based learning program, AETCOM (attitudes, ethics and communication), it contained 27 modules to be covered beginning from first year of training to final year of undergraduate training [28]. Clinical expertise does not ensure communication expertise. Therefore, scope of AETCOMs should be extended beyond undergraduate training. Without addressing receiver or family’s concerns and their perspective, efforts to improve communication among trainees will be incomplete. Future studies should focus on improving communication skills among trainees along with family satisfaction questionnaire as a possible yardstick for complete evaluation of communication process.

### Strengths and limitations

The pan India survey helped us to know perspective of trainees beyond geographical boundaries. Open ended questions gave the trainees liberty to share their experience which was the strength of our study.

As this was an online survey it had limitations due to study design. In analyzing the responses, we used content analysis method. We are unsure about the coherence between trainee’s perspective and our interpretation. In-person interview would have helped in knowing the trainee’s perspective in detail and different themes could have emerged. However, including open ended questions helped us to fulfil this lacuna. Designing of questionnaire using Likert scale could have helped in grading difficulty in counselling.

We urge the policy makers and various critical care societies to understand the need of the hour to emphasis on communication training. This could be implemented by including mandatory communication training in the existing curriculum and as a prerequisite before appearing for exams. Mentors should take the lead and train the aspiring intensivists to acquire these skills. To imbibe effective communication skills, conducting workshops, using simulation based teaching and supervised learning are the possible teaching learning methods.

## Conclusion

This survey helped to understand the level of knowledge, attitudes and practices of communication among ICU trainees. The formal training in communication was available only in 50% of participants. This survey demonstrated knowledge of communication skills is not adequate among trainees. With increasing complexities in a day-to-day practice empowering trainees with this essential skill is necessary. This effort will go a long way to improve and strengthen the doctor-patient bond.

## Supplementary Information

Supplementary Material 1

## Data Availability

The dataset used and/or analysed during the current study are available from the corresponding author on reasonable request.

## Abbreviations

AETCOM: Attitudes, Ethics and Communication
EOLC: End of life care
IAPC: Indian Academy of Palliative Care
ICU: Intensive Care Unit
IEC: Institutional Ethics Committee
ISCCM: Indian Society of Critical Care Medicine
OSCE: Objective Structured Clinical Examination
SPIKES: Setting up, Perception, Invitation, Knowledge, Emotions with Empathy, and Strategy or Summary
S-P-w-ICE-S: Setting, Perception, Warning call and pause, Information, Clarifying and dealing with Emotions, Strategy and Summary
VALUE: Validate, Assess, Listen, Understand, and Express
