# Supplementary material for: To assess the level of knowledge, attitude, and practice of communication among ICU trainees across India, TALK ICU SURVEY
Source: BMC Med Educ. 2025 Oct 17;25:1433. doi: 10.1186/s12909-025-07977-z (PMC12532392; doi:10.1186/s12909-025-07977-z)
Supplement: Supplementary file 1 — Supplementary Material 1 [file 12909_2025_7977_MOESM1_ESM.docx]

**Supplementary Table S1 Counselling details**

| **Formal training in communication n(%)** | |
| --- | --- |
| Yes | 72 (49.32) |
| No | 74 (50.68) |
| **Attend family meeting with consultant** | |
| Yes | 91 (62.33) |
| No | 55 (37.67) |
| **Opportunity to counsel independently** | |
| Yes | 141 (96.58) |
| No | 5 (3.42) |
| **Scheduled time for family meeting** | |
| Yes | 111(77.62) |
| No | 32 (22.38) |
| **Overcoming language barrier** | |
| Yes | 125 (85.62) |
| No | 21 (14.38) |
| **Frequency of counselling** |  |
| Once a day | 29 (19.86) |
| Twice a day | 34 (23.29) |
| Based on patient’s condition | 80 (54.79) |
| Others | 3 (2.05) |
| **Average time for counselling/patient** |  |
| 5 minutes | 19 (13.01) |
| 5-10 minutes | 71(48.63) |
| 10-15 minutes | 43(29.45) |
| >15 minutes | 13(8.9) |
| **Designated space for counselling** |  |
| Yes | 118(80.82) |
| No | 28(19.18) |
| **Availability of Audio-visual counselling** |  |
| Yes | 103 (70.55) |
| No | 43 (29.45) |
| **Routine recording of Audio-visual counselling available** |  |
| Selected patients  Yes  No | 34 (33.66)  57 (56.44)  10 (9.90) |

n (% percentage)

Supplementary Table S2 Hospital policies and practices

| **Code white policy** | Yes  No | 88 (60.27)  58 (39.72) |
| --- | --- | --- |
| **EOLC policy** | Yes  No | 87 (59.58)  59 (40.41) |
| **EOLC policy: need of the hour** | Yes  No | 141 (96.57)  5 (3.42) |
| **Do you have bereavement support** | Yes  No | 68 (46.57)  78 (53.42) |

EOLC-End of life care, n (% percentage)

Supplementary Table S3 Type of hospital and communication practices

| Parameter | Government Hospital  30 (20.54) | Private/ Corporate  96(65.75) | Private non profit  20(13.69) | p value |
| --- | --- | --- | --- | --- |
| Working hours  >48 hours | 28 (93.33) | 88(91.66) | 19 (95) | 0.859 |
| Formal training in communication | 17 (56.67) | 48 (50) | 7 (35) | 0.316 |
| Attending family counselling | 15 (50) | 63 (65.63) | 13 (65) | 0.294 |
| Opportunity for counselling | 30(100) | 91 (94.79) | 20 (100) | 0.260 |
| Scheduled time for counselling | 23(76.67) | 74 (79.57) | 14 (70) | 0.642 |
| Designated area for family meeting | 18 (60) | 81 (84.38) | 19 (95) | 0.003 |
| A-V counselling facility is available | 10 (33.33) | 76 (79.16) | 17 (85) | <0.001 |
| A-V Recording routinely used during family meetings if A-V counselling was available | 5 (50) | 39 (52) | 13 (81.25) | <0.001* |
| **Barriers for effective counselling** |  |  |  |  |
| Sociodemographic | 11 (36.67 | 34 (35.42) | 5 (25) | 0.681* |
| Language | 15(50) | 38 (39.58) | 10(50) | 0.483 |
| Burnout | 16(53.33) | 38 (39.58) | 9(45) | 0.408 |
| Gender of the treating doctor | 1(3.33) | 15(15.63) | 1(5) | 0.130* |
| Experience of the treating doctor | 8(26.67) | 23(23.96) | 7(35) | 0.590 |
| Education Level Of The Relatives | 27(90) | 70(72.92) | 18(90) | 0.057 |
| Severity of illness | 13(43.33) | 41(42.71) | 6(30) | 0.575 |
| Length of ICU stay | 7 (23.33) | 44(45.83) | 4(20) | 0.019* |
| **Presence of an EOLC policy** | 12 (40) | 63 (65.63) | 12 (60) | 0.044 |
| **Presence of CODE WHITE policy** | 11(36.67) | 65(67.71) | 12(60) | 0.010 |
| **Presence of Bereavement support system** | 13 (43.33) | 47 (48.96) | 8 (40) | 0.707 |

*Fischer’s exact test, n (% percentage), EOLC End of life care

Supplementary Table S4- Comparison based on formal training vs. No formal training in communication.

| Parameter | Formal training  72 (49.32) | No Formal training  74 (50.68) | p value |
| --- | --- | --- | --- |
| **Strategies used for counselling** |  |  |  |
| SPIKES | 10(13.89) | 10(13.51) | 0.947 |
| **Barriers in counselling** |  |  |  |
| Sociodemographic | 24 (33.33) | 26 (35.14) | 0.819 |
| Language | 27 (37.5) | 36 (48.65) | 0.174 |
| Burnout | 31(43.06) | 32 (43.24) | 0.982 |
| Gender | 9 (12.5) | 8 (10.81) | 0.750 |
| Experience | 14 (19.44) | 24 (32.43) | 0.074 |
| Education | 55 (76.39) | 60 (81.08) | 0.488 |
| **Faced Difficulty in breaking bad news** | 21 (29.17) | 36(48.65) | 0.016 |
| **Taken Informed Consent from the patient** | 63 (87.5) | 58(78.38) | 0.144 |
| **Communicated about Medical error** | 39 (54.17) | 21 (28.38) | 0.002 |

n(% percentage)

TALK ICU Survey for ICU Trainees- Questionnaire

ICU is a highly stressful environment. While taking care of patients, doctor has a dual responsibility to take care of patients relatives who are emotional, anxious, who expect empathy from the caregiver. There is inadequate training on communication skills in the critical care curriculum. The trainees may be naive to the situations encountered. This creates a big lacunae in Doctor- Patient relationship.

This survey is for trainees who are enrolled for the critical care course (DM/IDCCM/IFCCM/DrNB) and who have completed a minimum of 3 months of the training period.

This survey is designed to highlight various aspects of communication in ICU. We believe that communication is a skill like any other skills which can be learnt during the curriculum with formal training.

The information obtained from the survey will help in addressing various barriers to the effective communication.

The participation in survey is completely voluntary. The data collected is anonymous and ensures confidentiality of the participants. Participation in the survey is considered as a consent to use the data.

* Indicates required question

1. I have read the above information and I am willing to participate in a survey *

*Mark only one oval.*

Yes

No

# Survey

1. Age (years) *
2. Gender *

*Mark only one oval.*

Male

Female

Others

1. Years of experience in Critical care * *Mark only one oval.*

<=1 yrs

>1-5 yrs

>5-10 yrs

>10 yrs

1. Postgraduation in which speciality ? * *Mark only one oval.*

General Medicine

Anaethesiology

Pulmonary medicine

Emergency Medicine

1. Enrolled for which critical care course ? * *Mark only one oval.*

IDCCM

IFCCM

DrNB/DNB

DM

Fellowship in Critical Care

1. What is the duration of your training?

*Mark only one oval.*

>3 months to 6 months

6 months to 1year

1 year to 2 years

2-3 years Other:

1. Type of Hospital you are working in? * *Mark only one oval.*

Government/ Medical college

Private Hospital/Non Pro t

Private /Corporate Hospital

1. Number of patients seen per day * *Mark only one oval.*

<10

10-20

20-30

>30

1. Working hours per week *

*Mark only one oval.*

<48 hrs

>48 hrs

1. Are you formally trained in ICU Communication during Intensive care training ? *

*Mark only one oval.*

Yes

No

# Doctor -Patient communication

1. Do you attend daily family meetings with the consultant? *

*Mark only one oval.*

Yes

No

1. Do you get the opportunity to counsel the relatives independently? *

*Mark only one oval.*

Yes

No

1. Do you have scheduled time for family meeting?

*Mark only one oval.*

Yes No

1. If you are not familiar with patients language , do you take help of a translator while counselling ? *

*Mark only one oval.*

Yes

No

1. What is the frequency of counselling per day? *

*Mark only one oval.* once in a day twice a day

Based on patient's condition Other:

1. Average Time taken for counselling for each patient? *

*Mark only one oval.*

5minutes

5-10 minutes

10-15 minutes

> 15 minutes

1. Is counselling done in the designated area for the counselling? *

*Mark only one oval.*

Yes

No

1. Is the audio-visual counselling (ensures audio visual recording of the counselling) facility available in your hospital ? *

*Mark only one oval.*

Yes

No

1. If yes, Is the audio visual recording of the counselling done routinely?

*Mark only one oval.*

Yes

No

For Selected patients

Other:

1. What are the strategies you follow during counselling? (< 50 words) *
2. What are the barriers in counselling? *

*Check all that apply.*

Gender

Language

Socioeconomic status

Education level of the relatives

Severity of illness

Experience of the treating doctor

Length of ICU stay Burnout Other:

1. Do you find difficulty in breaking bad news? *

*Mark only one oval.*

Yes

No

1. If yes , kindly elaborate (<50 words)
2. Informed consent - Do you explain all the complications pertaining to a procedure while taking consent? *

*Mark only one oval.*

Yes

No

Sometimes

Depends upon type of intervention

Other:

1. Have you taken consent from a patient for a particular procedure if patient's condition allows to do so ? *

*Mark only one oval.*

Yes

No

1. Have you counselled the relatives about a medical error anytime? *

*Mark only one oval.*

Yes

No

1. What are the reasons for interdisciplinary conflicts? (Doctor-Doctor, Doctor-Nurse, Doctor-Administration) (<50 words) *

# End of Life Care (EOLC)

11

1. Do you have a EOLC (End of Life Care) policy in your hospital? *

*Mark only one oval.*

Yes

No

1. Do you think EOLC policy is need of the hour? *

*Mark only one oval.*

Yes

No

1. Do you have Bereavement support system? (emotional support to the family ) *

*Mark only one oval.*

Yes

No

# Patient /Family satisfaction

1. What are the factors influencing patient /family satisfaction ? *

*Check all that apply.*

Visiting hours

Counselling by Senior most faculty

Paramedical staffs interaction with the family

Lack of empathy among caregivers

Lack of administrative support

Outcome of the patient Other:

1. Do you have a policy to activate "Code White" (Violence against doctors) in your hospital ? *

*Mark only one oval.*

Yes

No

1. Has the Covid-19 pandemic influenced the Doctor- Patient relationship? (<50 words) *
2. What are your suggestions to improve "Communication skills" in ICU (<50 words) ? *

This content is neither created nor endorsed by Google.

[Forms](https://www.google.com/forms/about/?utm_source=product&utm_medium=forms_logo&utm_campaign=forms)
